# Compound Opening Arrow Mixture exerts anti-tumor effects in a mouse model of breast cancer

**DOI:** 10.1038/s41598-020-64561-9

**Published:** 2020-05-18

**Authors:** Zhen Zhou, Yanfang Peng, Wang Ai, Qi Li, Taisheng Ye, Chaoyan Wu, Haoliang Ke, Xiuping Wang, Yingwen Zhang

**Affiliations:** 1 Deparment of Traditional Chinese Medicine, Zhongnan Hospital of Wuhan University, Wuhan, China; 2 Shuguang Hospital Affiliated to Shanghai University of Traditional Chinese Medicine, Shanghai, China

**Keywords:** Experimental models of disease, Breast cancer

## Abstract

Compound Opening Arrow Mixture (COAM) has demonstrated therapeutic effects in patients with breast cancer. We explored the underlying molecular mechanisms of COAM using a mouse model of breast cancer. Luciferase-labeled 4T1-Luc2 cells were inoculated into the breast pad of BALB/c-nu mice, which were divided into model group (saline), COAM (6 g/ml high-dose, 3 g/ml medium-dose, and 1.5 g/ml low-dose) groups, and low-molecular-weight heparin (LMWH, 1500 U/Kg) group. The number and distribution of 4T1-luc2 tumors were measured by an *in vivo* imaging system. Tumor cell apoptosis was measured through TUNEL and quantitating the expression of Caspase-3 mRNA and protein. Compared with the model group, *in vivo* tumor growth was lower in the LMWH- and COAM-treated groups. Tumor apoptosis was time-dependent and dose-dependent, as shown by a higher TUNEL apoptotic index and higher Caspase-3 mRNA and Caspase-3/cleaved-Caspase-3 proteins levels on the 14th day than the 7th day. The COAM high-dose group had the highest apoptotic index and the most activation of Caspase-3. Collectively, COAM significantly inhibits the growth of 4T1-luc2 breast cancer in mice and induces tumor apoptosis by activating Caspase-3, which provides a preliminary explanation of therapeutic effects of COAM.

## Introduction

Breast cancer is one of the most common malignant diseases that threaten women’s health. It ranks first in the incidence of female malignancies, with approximately 279,000 new cases diagnosed each year^1,2^. In China, the incidence of breast cancer increases at a rate of about 2% per year, and breast cancer causes an average annual mortality rate exceeding 70, 000 patients^1,2^. Surgery, radiotherapy, chemotherapy, endocrine therapy, and targeted therapy are the major treatment strategies for breast cancer. Although the efficacy of these treatments has improved in recent years, recurrence and metastasis of breast cancer are still the main causes of death^3^. Therefore, new strategies for the effective control of the metastatic breast cancer are urgently needed.

Traditional Chinese medicine also plays a critical role in the treatment of breast cancer. Based on more than 30 years of clinical experience and the theory of traditional Chinese medicine in tumor suppression and immune enhancement^4,5^, we developed the Compound Opening Arrow Mixture (COAM)^6^. COAM has been used as treatment for patients with middle-late stage breast cancer, and clinically sound therapeutic effects have been achieved in these patients in our hospital.

To investigate the mechanisms underlying the therapeutic effects of COAM in breast cancer, we established a mouse model of breast cancer using a highly tumorigenic and invasive, luciferase-labeled mouse breast cancer cell line 4T1-Luc in this study. An *in vivo* fluorescence imaging system was used to monitor the growth of breast cancer in tumor-bearing mice at different time points after oral administration of COAM and low-molecular-weight heparin (LMWH), which is a compound with proven anti-tumor activity. The effect of COAM at different doses on the apoptosis of transplanted tumor cells was evaluated and compared with that of LMWH. Our study suggests that like LMWH, COAM exerts its anti-tumor activity by promoting the apoptosis of transplanted tumor cells in mice.

## Methods

### Cell culture

Luciferase-labeled mouse breast cancer cells 4T1-luc were obtained from Shanghai Keyuandi Biotechnology Co., Ltd, Shanghai, China. Cells were cultured in RPMI 1640 medium supplemented with 10% fetal bovine serum (Gibco), 100 μg/ml penicillin and 100 μg/ml streptomycin, and were maintained in an incubator with 5% CO_2_ at 37 °C.

### Compounds for animal treatments

Compound Opening Arrow Mixture (COAM) was made from traditional Chinese herbs. It consisted of opening arrow (7.14% by weight), Tuckahoe (5.71%), astragalus membranaceus (4.29%), lycopodium clavatum (7.14%), loofah sponge (7.14%), kelp (7.14%), bulbus Fritillariae Thunbergii (7.14%), hedyotis diffusa (7.14%), rhizoma sparganic (7.14%), hirudo (4.29%), curcuma (5.71%), prunella vulgaris (7.14%), curcuma (5.71%), Sargent gloryvine (5.71%), dandelion (5.71%), saponin (5.71%) and was provided by the Department of Chinese Pharmacy of Zhongnan Hospital of Wuhan University. The ground components of these traditional Chinese herbs were mixed and dissolved in heated distilled water to prepare different concentrations of COAM: low-dose, 1.5 g/ml; medium-dose, 3 g/ml; and high-dose, 6 g/ml. Low molecular weight heparin was purchased from the Department of Western Pharmacy of Zhongnan Hospital of Wuhan University.

### Breast cancer mouse model

Female specific-pathogen-free (SPF)-grade BALB/c-nu mice (weighing 20 g ± 2 g, 6 weeks old) were purchased from Beijing Weitong Lihua Experimental Animal Technology Co., Ltd. [License No.: SCXK2016-0006]. Mice were raised in the Animal Experimental Center of Huazhong Agricultural University in Wuhan, China. All experiments involving the use of animals in the protocol (HZAUMO-2018-020) were approved by the ethical committee of Huazhong Agricultural University. All methods were performed in accordance with the relevant guidelines and regulations.

After the bioluminescent labeling activity of 4T1-luc cells reached 99% or more, cells in the logarithmic growth phase were prepared in a suspension at a concentration of 1×10^7^/mL for inoculation. Ten mice were randomly selected as the normal control group (no cancer cell injection). The remaining mice were inoculated with 20 μl of suspended 4T1-luc cells into the right breast pad to prepare a breast cancer mice model. On the first day after the cells were inoculated, the inoculation efficiency was measured using a small animal *in vivo* imaging system (IMS Image Analysis System; Wuhan Hualianke Biotechnology Co., Ltd., China)^7,8^. The detected total photon number (p/s/mm^2^) indicates the number of tumor cells inoculated into the breast pad. Mice with uniform inoculation were included in the experiment, and 10 mice were randomly assigned to each group.

After all mice were successfully inoculated, they were randomly divided into 5 groups: model group (no drug), low-molecular-weight heparin (LMWH) group, COAM-high, COAM-medium, and COAM-low groups. On the next day after successful modeling, mice in the COAM (high, medium and low doses) groups were given 1.5 g/ml, 3 g/ml, 6 g/ml of COAM by gavage once daily. Mice in the low-molecular-weight heparin group were given intraperitoneal injection of low-molecular-weight heparin (1500 U/Kg) once a day. Mice in the model group and normal control group were given equal doses of saline once daily by gavage. Five mice were randomly selected from each group on the 7th and 14th day after drug administration, and the tumor tissues were removed for analyses of apoptosis.

### Tumor *in vivo* imaging and measurement

The number, size, and distribution of the transplanted tumors were observed by the small animal living imaging system (IMS Image Analysis System) on the next day after inoculation, the next day after successful modeling, the seventh day after drug administration, and the 14th day after drug administration. Tumor size is expressed as the total number of photons (p/s/mm^2^) of the detected tumor cells. After anesthetization with 3% isoflurane for 5 min, the mice were intraperitoneally injected with 150 mg/kg substrate Luciferin Potassium Salt (3 mg/mouse; Goldbio, USA). After 10 minutes, the total luminescence of the tumor cells was measured. The long and short diameters of the tumors were measured with a caliper, and tumor volumes were calculated with the following formula: V = (a×b ^2^)/2, where a indicates the long diameter, and b indicates the short diameter.

### TUNEL assay for tumor cell apoptosis

The TUNEL (Terminal deoxynucleotidyl transferase dUTP nick end labeling) assay was performed with a TUNEL apoptosis detection kit (chromogenic method) following the manufacturer’s instructions. The *in situ* TUNEL images of the transplanted tumor tissues with cell pyknosis, chromatin condensation block or edge set, brown, yellow, or brownish yellow staining were indicative of apoptotic cells. At each time point, three mice from each group were taken for the TUNEL assay. The number of apoptotic cells in three observation fields was counted for each slice, and the apoptotic index was calculated with the following formula: Apoptosis index = TUNEL positive cells / total number of cells × 100%.

### Quantitative PCR assay for determining caspase-3 mRNA

Total RNA was extracted from tumor tissues with Trizol reagent (Ambion) following the manufacturer’s instructions. Complementary DNA (cDNA) was reverse transcribed using a reverse transcription kit (TAKARA). PCR primers (Table 1) were synthesized by Nanjing Kingsley Biotechnology Co., Ltd. Using GAPDH as an internal reference gene, real-time quantitative PCR (RT-qPCR) assay was performed to detect the expression of Caspase-3 mRNA with the SYBR Green PCR Kit (KAPA Biosystems) and a Real-Time System (BIO-RAD). The reaction conditions were as follows: 95 °C for 3 min; and 39 cycles of 95 °C for 5 sec, 56 °C for 10 sec, 72 °C for 25 sec. Each sample had 3 replicates. The data were analyzed with qBase PLUS and the relative mRNA expression of the target gene was calculated using the 2^-ΔΔCt^ method.

**Table 1:** The sequences of PCR primers used in this study.

Primer name	Sequences (5’- 3’)	Amplified fragment size (bp)
Caspase-3-F	TGGGACTGATGAGGAGA	125
Caspase-3-R	ACTGGATGAACCACGAC	
GAPDH-F	CCTTCCGTGTTCCTAC	152
GAPDH-R	GACAACCTGGTCCTCA	

### Western blot assay for measuring cle-caspase-3 and caspase-3 proteins

At each time point, three mammary gland tissues of randomly selected mice from each group were ground and lysed with radioimmunoprecipitation (RIPA) buffer (Bioswamp). After centrifugation, the supernatant was collected and the protein concentration was determined with the bicinchoninic acid assay (BCA) protein concentration assay kit (Enhanced) (Bioswamp). Ten µg of each sample was loaded and separated using sodium dodecyl sulfate polyacrylamide gel electrophoresis (SDS-PAGE). After transferring protein to the polyvinylidene difluoride (PVDF) membrane, samples were blocked with 5% bovine serum albumin (BSA) blocking solution at room temperature for 1 h. The membranes were incubated with the primary antibody overnight at 4 °C (Caspase-3 (antibody species rabbit): abcam, article number ab4051, 1:500 dilution; Cle-caspase-3 (antibody species rabbit): CST, article number 9661, 1:1,000 dilution; GAPDH (antibody species rabbit): CST, article number 2118). After washing with PBST (PBS containing 0.5% Tween-20), the membranes were incubated with horseradish peroxidase (HRP) conjugated secondary antibody (Goat Anti-Rabbit IgG (antibody species Goat); bioswamp, article number PAB160011, goat anti rabbit antibody in 5% skim milk, 1:10,000 dilution) for 1 h at room temperature, and washed with PBST 3 times, 5 min each time. The membrane was further processed with a chemiluminescence reagent (Millipore) on a fully automatic chemiluminescence analyzer (Shanghai Tianon), and the relevant stripe gray values were read and quantified by TANON GIS software (Shanghai, China).

### Statistical analysis

Data were analyzed by SPSS 21.0 statistical software (IBM, USA), and measurement data were expressed as mean ± SD (standard deviation). Statistical analyses were performed using one-way ANOVA (analysis of variance). A *p* < 0.05 was considered statistically significant.

## Results

### Administration of COAM significantly reduced the number of transplanted tumor cells

**Figure 1:**
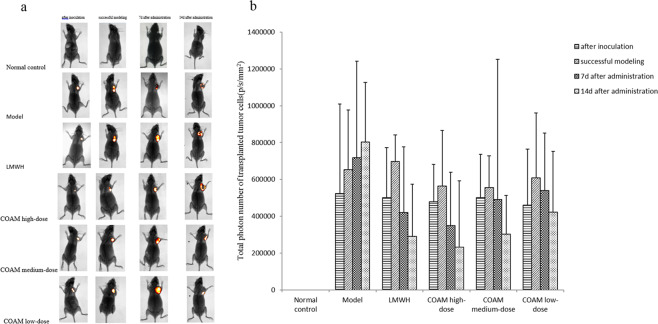
*In vivo* imaging results of transplanted breast cancer cells 4TI-Luc in mice. **(a-b)** Female BALB/c-nu mice were inoculated with luciferase-labeled mouse breast cancer cells 4T1-luc. Mice were mock-treated with saline, were treated with low-molecular-weight heparin, or various doses of Compound Opening Arrow Mixture (COAM) after successful modeling. The growth of transplanted tumor cells was observed using a small animal *in vivo* imaging system on the 1st day after successful modeling and on the 7th and 14th day after drug administration. **(a)** Representative *in vivo* images of mice on the 7th day after drug administration. **(b)** Summarized data on total photon number of transplanted tumor cells (p/s/mm2) in the indicated groups at specified time points. Normal control group, mice without inoculation of tumors and treatments; Model group, mice with inoculation of tumors and treatments with saline; LMWH group, mice with inoculation of tumors and treatments with 1500 U/Kg low-molecular-weight heparin once a day by gavage; COAM high-dose group, mice with inoculation of tumors and treatments with high-dose of COAM (6 g/ml) once daily by gavage; COAM medium-dose group, mice with inoculation of tumors and treatments with medium-dose of COAM (3 g/ml) once daily by gavage; COAM low-dose group, mice with inoculation of tumors and treatments with low-dose of COAM (1.5 g/ml) once daily by gavage. n = 5 for each group.

After 4T1-luc cells were inoculated into the BALB/c-nu mice, the cells were imaged immediately after inoculation, and all mice were illuminated at this inoculation point. In addition, the growth of transplanted tumor cells was measured on the 1st day after successful modeling (7 days after tumor cell inoculation)^9^, and on the 7th and 14th day after drug administration. Because of the overlapping location of breast tissue and lung tissue, it is impossible to accurately determine whether cancer metastasis has occurred from the perspective of live imaging. Thus, we judged tumor growth by the photon number obtained during live imaging. As the number of observation days increased, the total number of photons (Fig. 1a) in the model group increased, and tumor cell-derived luminescence intensity also increased. As shown in Fig. 1b, the total number of photons in the treatment groups (LMWH and COAM-treated groups) on the 1st day after the successful modeling was the greatest, and it decreased on the 7th and 14th days, which was significantly different from the model group receiving no drug.

The COAM-treated groups had a drug dose-dependent effect on the reduction of tumor cells-derived photons. On the 7th day after COAM administration, the total photon number in the COAM high-dose group was significantly lower than that in the COAM medium-dose group and COAM low-dose group. There was no significant difference between the COAM high-dose group and the LMWH group. On the 14th day after drug treatment, a positive correlation between the dose of COAM and reduction of photon number was observed in the COAM-treated groups. Notably, at this time point, the COAM high-dose group demonstrated smaller photon number, compared with the LMWH group, which indicated that COAM at a high concentration had better therapeutic effects than low-molecular-weight heparin in our assay in terms of eliminating tumorigenic cells.

### COAM treatments significantly reduced the volume of transplanted tumor in nude mice

**Figure 2:**
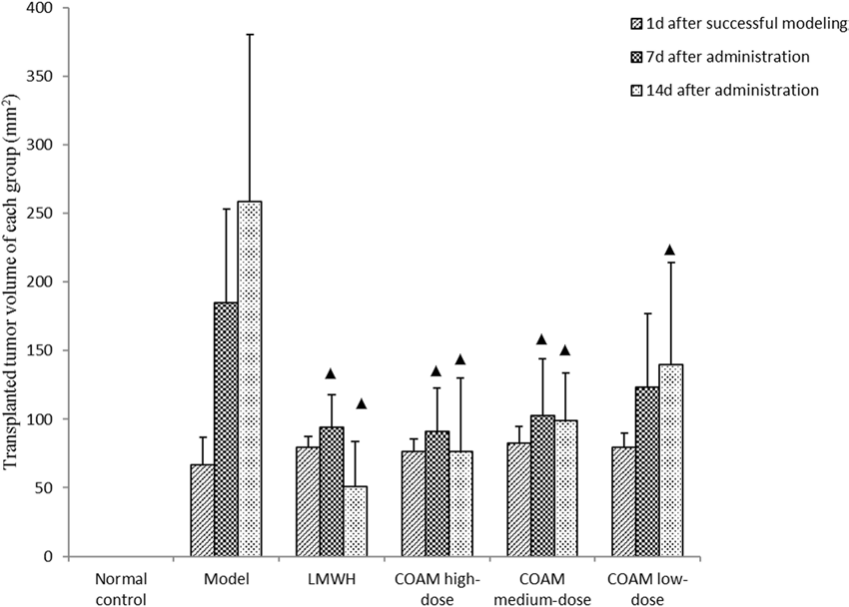
COAM treatments significantly reduced the volume of transplanted tumor in nude mice. Results shown are tumor volumes at indicated time points in specified groups. n = 5 for each group. ▲*p* < 0.05, compared with the model group.

As shown in Fig. 2, on the first day after successful modeling, there was no significant difference in the volume of transplanted tumors among the groups. On the 7th day after drug administration, the tumor volume of the model group was significantly increased, whereas the LMWH group and COAM-treated groups had significantly smaller tumor volumes than the model group. However, we observed no significant differences among the various treatment groups.

On the 14th day after drug administration, the LMWH group and COAM-treated groups demonstrated a significant reduction in tumor volume than the model group. Although the LMWH group had a lower mean value of tumor volume than the COAM high-dose group, the differences between the LMWH group and the COAM high-dose group or the COAM medium-dose group were not statistically significant (Fig. 2).

### TUNEL assay demonstrated that COAM administration dose-dependently promoted the apoptosis of tumor cells in mouse breast tissues

**Figure 3:**
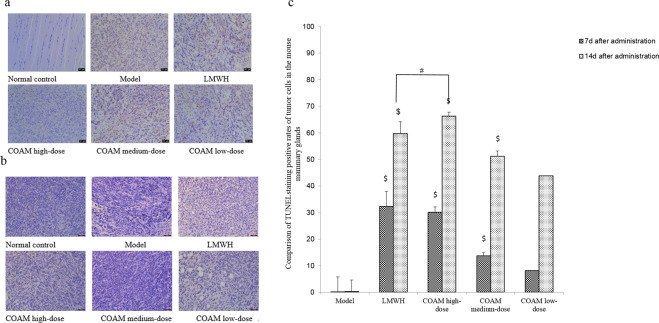
TUNEL assay demonstrated that COAM administration dose-dependently promoted the apoptosis of tumor cells in mouse breast tissues. **(a-b)** Representative TUNEL assay images of tumor cells in mouse mammary glands in the specified groups on the 7th day (a) and the 14th day (b) after drug administration. Scale bar, 50 μm. **(c)** Comparison of TUNEL staining positive rates of tumor cells in the mouse mammary glands. n = 5 for each group. ^#^*p* < 0.05, compared with the LMWH group; ^$^*p* < 0.05, compared with the COAM low-dose group.

Apoptosis of tumor cells in breast tissue samples was detected by the TUNEL assay. As shown in Fig. 3, there were almost no apoptotic cells in the normal control group and the model group. At each time point, the apoptotic index of COAM-treated (high, medium and low dose) groups and the LMWH group were significantly higher than the model group. For each treatment group, the apoptotic index at the 14th day was higher than that on the 7th day. Remarkably, COAM administration resulted in a dose-dependent effect in promoting tumor cell apoptosis, as revealed by the highest apoptotic index in the high-dose group and the lowest apoptotic index in the low-dose group. Compared with the LMWH group, the COAM high-dose group was not significantly different in the apoptotic index on the 7th day. However, the apoptotic index on the 14th day was higher in the COAM high-dose group than the LMWH group (Fig. 3).

### COAM administration significantly increased Caspase-3 mRNA in mouse breast tissues

**Figure 4:**
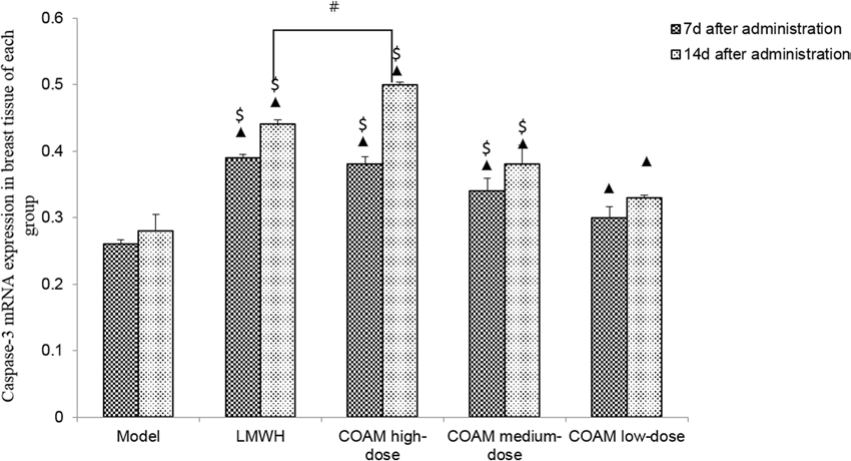
COAM administration significantly increased Caspase-3 mRNA levels in mouse breast tissues. Data shown are the summarized results comparing Caspase-3 mRNA expression in breast tumor cells in the specified groups. n = 5 for each group. ▲*p* < 0.05, compared with the model group; ^#^*p* < 0.05, compared with the LMWH group; ^$^*p* < 0.05, compared with the COAM low-dose group.

We evaluated mRNA levels of Caspase-3, a key regulator of apoptosis, in tumor cells from the breast tissues in mice. For each treatment group, Caspase-3 mRNA at the 14th day was higher than that on the 7th day after drug treatment (Fig. 4). Compared with the model group, Caspase-3 mRNA was significantly increased in the COAM-treated (high, middle, low dose) groups and LMWH group. Caspase-3 transcript levels in the COAM-treated groups showed a significant dose-dependent increase, as the COAM high-dose group had the highest levels of caspase-3, whereas the COAM low–dose group had the lowest level (Fig. 4). However, there was no significant difference between the LMWH group and COAM high-dose group.

On the 14th day after drug administration, compared with the model group, the expression of caspase-3 mRNA was significantly increased in the COAM-treated (high, middle, low dose) groups and LMWH group. Notably, the COAM high-dose group had slightly more caspase-3 mRNA than the LMWH group. Similarly, we observed a positive correlation between COAM dose and caspase-3 mRNA level in tumor cells in the COAM-treated group at 14 days after drug administration (Fig. 4).

### COAM administration significantly increased the Caspase-3 and cleaved caspase-3 proteins in mouse breast tissues

**Figure 5:**
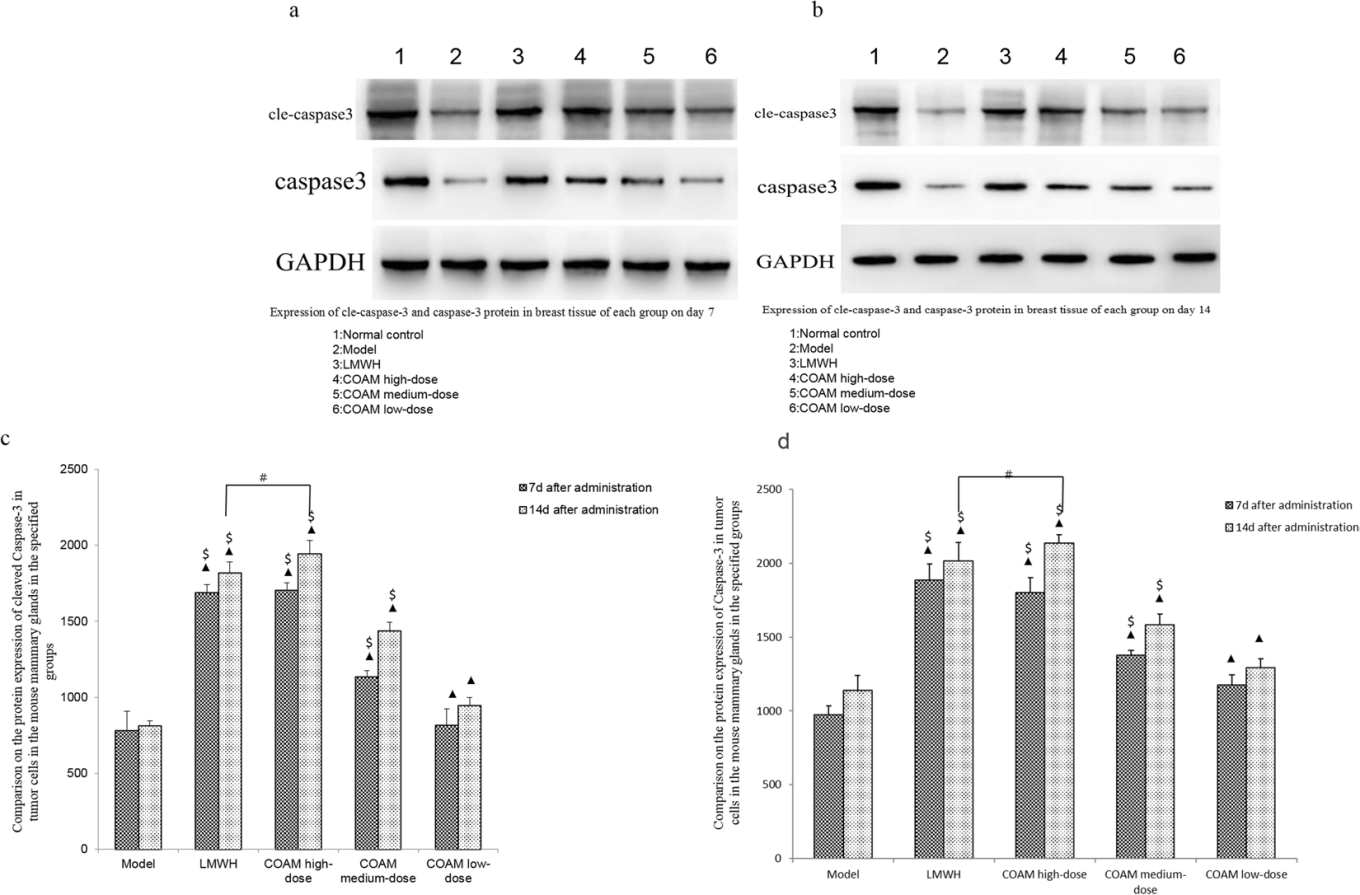
COAM administration significantly increased Caspase-3 and cleaved Caspase-3 protein in mouse breast tissues. **(a-b)** Representative western blot images of Caspase-3 and cleaved caspase-3 in tumor cells in mouse mammary glands in the specified groups on the 7th day (a) and the 14th day (b) after drug administration. **(c-d)** Comparison of protein expression of cleaved Caspase-3 (c) and Caspase-3 (d) in tumor cells in the mouse mammary glands in the specified groups. n = 5 for each group. ▲*p* < 0.05, compared with the model group; ^#^*p* < 0.05, compared with the LMWH group; ^$^*p* < 0.05, compared with the COAM low-dose group.

We examined caspase-3 and cleaved caspase-3 (cle-caspase-3) protein levels in transplanted tumor cells. For each treatment group, caspase-3 and cle-caspase-3 protein levels on the 14th day were higher than that on the 7th day after drug treatment (Fig. 5). On the 7th day, compared with the model group, the expression of caspase-3 and cle-caspase-3 protein was significantly increased in the COAM-treated (high, middle, low dose) groups and LMWH group. Consistently, the protein levels of caspase-3 and cle-caspase-3 in the COAM-treated groups showed a significant dose-dependent increase, as the COAM high-dose group had the highest levels, while the COAM low–dose group had the lowest levels (Fig. 5). However, there was no significant difference between the LMWH group and COAM high-dose group. On the 14th day after drug administration, we observed similar trends of caspase-3 and cle-caspase-3 protein expression of what we found on the 7th day after drug administration (Fig. 5). It is worth noting that caspase-3 and cle-caspase-3 the protein levels in the COAM high-dose group on the 14th day was slightly higher than that in the LMWH group.

## Discussion

The theory of Chinese medicine suggests that the occurrence and development of breast cancer result from long-term interactions between the internal and external factors. The deficiency of vital energy is due to the body’s weak immune function, and pathogens can take advantage of it for invading the host. The factors “deficiency”, “poison”, “phlegm” and “stasis” themselves and the interactions among these factors facilitate the occurrence, development, and degeneration of breast cancer^10,11^. Most breast cancer patients are characterized by the weakness of righteousness, and affected by the intrinsic effects of pathogenic toxin and blood stasis.

Therefore, the focus of breast cancer treatment lies in efficiently promoting the blood circulation, detoxifying, and dissolving. The COAM was developed based on resolving the pathological features of “deficiency”, “poison”, “phlegm”, and “stasis” in breast cancer. It is composed of opening arrow, tuckahoe, astragalus membranaceus, lycopodium clavatum, loofah sponge, kelp, bulbus fritillariae thunbergii, hedyotis diffusa, rhizoma sparganic, hirudo, curcuma, and prunella vulgaris. Tuckahoe and astragalus membranaceus help the release of toxin, the clear of damp, and promote diuresis. Bulbus Fritillariae Thunbergii, kelp, opening arrow, hedyotis diffusa and prunella vulgaris contribute to heat-clearing and detoxifying, and resolution of hard lumps. Lycopodium clavatum and loofah sponge conduce to expelling wind and dredging collat. Rhizoma sparganic, hirudo and curcuma break the blood stasis. All of these components jointly contribute to the therapeutic effect of COAM in promoting blood circulation, detoxifying and dissolving in patients with breast cancer.

From the perspective of pharmacological mechanisms, more and more basic studies have found that Chinese herbal medicine plays a clear role in the treatment of breast cancer^12^^.^ For example, hedyotis diffusa can reduce the cytotoxic effect of chemotherapy drugs on breast cancer cells^13^. Triterpenic acid, an active ingredient in tuckahoe, has cytotoxic and apoptosis-inducing activities against tumor cells, and further studies have found that it induces apoptosis through the Caspase 3/7-involved mitochondrial pathway^14,15^. Moreover, the effective active monomer β-elemene in curcuma can inhibit the migration and invasion of 4T1 mouse breast cancer cells by heparanase^16^. In addition, Zhao et al^17^. found that Ruanjian Sanjie decoction (made from prunella vulgaris) can induce apoptosis of breast cancer cells and exert anti-tumor activity. Furthermore, clinical case studies have found that when combined with paclitaxel, prunella vulgaris increased pathological complete response (pCR) rate and overall survival (OS) rate of patients with breast cancer. Besides, prunella vulgaris can prevent side effects caused by chemotherapy, such as neutropenia fever and chemotherapy-induced anemia^18^.

Our study demonstrated that the Compound Opening Arrow Mixture has an anti-tumor effect on inhibiting the growth and promoting apoptosis of mouse breast cancer 4T1-luc2. We not only explored the mechanism of a clinically effective Chinese medicine prescription, but also used the latest *in vivo* imaging technology (bioluminescence imaging) to measure the growth of tumor cells. The technology is intuitive, real-time and sensitive, and allows for dynamic observation^19^. It can obtain a series of data repeatedly in the same individual, which helps to eliminate individual differences, reduce the number of animals, conform to the “3 R” principle^20^.

The total photon number and distribution of transplanted tumor cells in mice after inoculation, successful modeling, and on the 7th and 14th day after drug administrations were measured by *in vivo* imaging. We compared the anti-tumor effects of various treatments in breast tumor-bearing mice. Heparin and its derivatives are potential anti-metastatic agents with good biocompatibility in many metastatic cancers like breast cancer^21–23^. Our study also confirmed the role of LMWH in potent suppression of the metastatic breast tumor cells 4T1-luc2. COAM exerted anti-tumor effects in a dose dependent manner, as COAM at high-dose (6 g/ml) had the highest suppressive ability, while COAM at low-dose (1.5 g/ml) demonstrated the lowest suppressive ability in *in vivo* tumor growth. Notably, in terms of the photon numbers, COAM at high-dose was more effective than LMWH at 1500 U/Kg in our animal model, as the mean value of the photon number in the COAM high-dose group is smaller than that in the LMWH group. However, there was no statistical significance between these two groups regarding the tumor volume. This is probably due to the lower sensitivity of using tumor volumes to indicate tumor apoptosis, when compared with the approach of measuring photon number. Further, TUNEL was used to detect the apoptosis of tumor cells in the breast tissue samples, and similar results on the trend of apoptosis were obtained. We verified that COAM can promote apoptosis of tumor cells in breast cancer-bearing mice, and we observed a correlation between the anti-tumor effect and apoptotic index had been identified in the LMWH and COAM treated groups.

Caspase-3 is a key factor in various apoptotic signaling pathways. Activation of caspase-3 is the hallmark event in inducing apoptosis in tumor cells^24^. In this study, we examined caspase-3 and cle-caspase-3 expression levels. Similar trends of mRNA expression and protein expression of caspase-3 were obtained in COAM-treated groups. The protein levels of both caspase-3 and cle-caspase-3 in the COAM high-, medium- and low-dose groups were higher than those in the model group, especially on the 14th day after the drug administration. Of note, the COAM high-dose group demonstrated higher caspase-3 and cle-caspase-3 protein levels than the LMWH group, which potentially further supports that COAM at 6 g/ml is more effective than LMWH at 1500 U/Kg for tumor elimination in our animal model. Taken together, COAM exerts anti-tumor effects through the activation of caspase-3 and the induction of apoptosis to inhibit tumor proliferation.

In summary, COAM exerts prominent anti-proliferative effects on the metastatic breast cancer *in vivo* through the induction of caspase-3 activation-mediated apoptosis in tumor cells. Although the main components responsible for the anti-tumor effect of COAM, and the in-depth mechanism of action of each drug component deserve further investigation in future, our work provides a preliminary explanation on the basis of the clinical therapeutic effects of COAM in treating patients with metastatic breast cancer.

### Ethical approval

All experiments involving the use of animals in the protocol (HZAUMO-2018-020) were approved by the ethical committee of Huazhong Agricultural University. All methods were performed in accordance with the relevant guidelines and regulations.

## Data Availability

The datasets generated during and/or analysed during the current study are available from the corresponding author on reasonable request.
