# Author Correction: Compound Opening Arrow Mixture exerts anti-tumor effects in a mouse model of breast cancer

**DOI:** 10.1038/s41598-022-22606-1

**Published:** 2022-11-15

**Authors:** Zhen Zhou, Yanfang Peng, Wang Ai, Qi Li, Taisheng Ye, Chaoyan Wu, Haoliang Ke, Xiuping Wang, Yingwen Zhang

**Affiliations:** 1 Deparment of Traditional Chinese Medicine, Zhongnan Hospital of Wuhan University, Wuhan, China; 2 Shuguang Hospital Affiliated to Shanghai University of Traditional Chinese Medicine, Shanghai, China

Correction to: *Scientific Reports* 10.1038/s41598-020-64561-9, published online 18 May 2020

The original version of this Article contained an error in Figure 1a, where 14d image did not display correctly.

The original Figure 1 and accompanying legend appear below.

**Figure 1:**
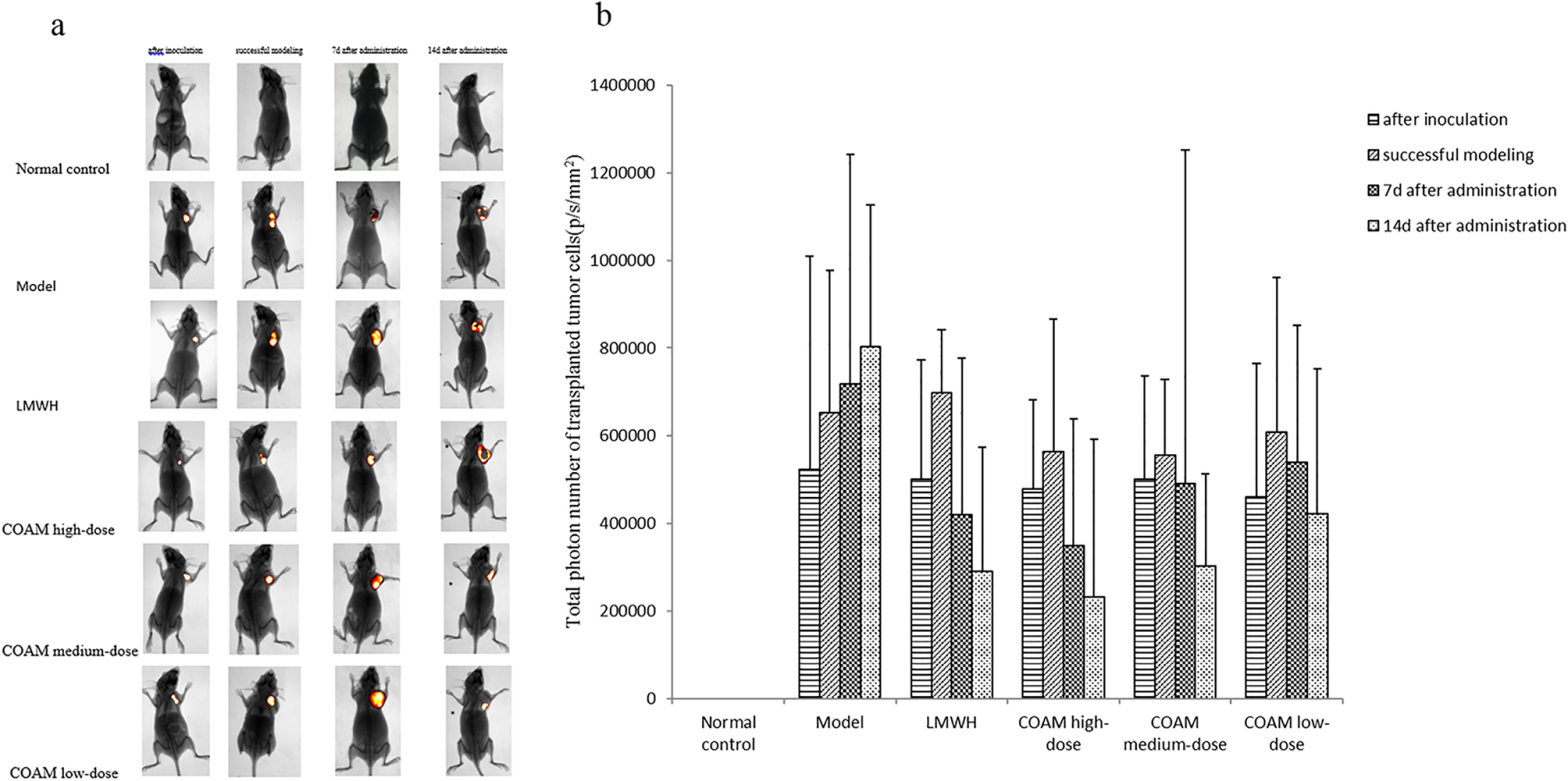
*In vivo* imaging results of transplanted breast cancer cells 4TI-Luc in mice. (**a**–**b**) Female BALB/c-nu mice were inoculated with luciferase-labeled mouse breast cancer cells 4T1-luc. Mice were mock-treated with saline, were treated with low-molecular-weight heparin, or various doses of Compound Opening Arrow Mixture (COAM) after successful modeling. The growth of transplanted tumor cells was observed using a small animal *in vivo* imaging system on the 1st day after successful modeling and on the 7th and 14th day after drug administration. (**a**) Representative *in vivo* images of mice on the 7th day after drug administration. (**b**) Summarized data on total photon number of transplanted tumor cells (p/s/mm2) in the indicated groups at specified time points. Normal control group, mice without inoculation of tumors and treatments; Model group, mice with inoculation of tumors and treatments with saline; LMWH group, mice with inoculation of tumors and treatments with 1500 U/Kg low-molecular-weight heparin once a day by gavage; COAM high-dose group, mice with inoculation of tumors and treatments with high-dose of COAM (6 g/ml) once daily by gavage; COAM medium-dose group, mice with inoculation of tumors and treatments with medium-dose of COAM (3 g/ml) once daily by gavage; COAM low-dose group, mice with inoculation of tumors and treatments with low-dose of COAM (1.5 g/ml) once daily by gavage. n = 5 for each group.

The original Article has been corrected.

